# Cross Cultural Analysis of Direct Employee Participation: Dealing With Gender and Cultural Values

**DOI:** 10.3389/fpsyg.2019.00723

**Published:** 2019-04-16

**Authors:** Marta Valverde-Moreno, Mercedes Torres-Jiménez, Ana M. Lucia-Casademunt, Yolanda Muñoz-Ocaña

**Affiliations:** ^1^Departamento de Gestión y Métodos Cuantitativos, Universidad Loyola Andalucía, Córdoba, Spain; ^2^Departamento de Métodos Cuantitativos, Universidad Loyola Andalucía, Córdoba, Spain; ^3^Departamento de Gestión Empresarial, Universidad Loyola Andalucía, Córdoba, Spain

**Keywords:** employees’ participation in decision making, PDM, perceived supervisor support, gender gap in PDM, national cultural values, European countries

## Abstract

The goal of this study is analyze the influence of perceived supervisor support (PSS) by employees at a micro level and the role of the cultural values of “power distance” and “masculinity” at a macro level on direct employee participation in decision-making (PDM). Furthermore, the influence of the gender of managers and employees is taken into account. The analysis is based upon the Sixth European Working Conditions Survey carried out by Eurofound in 2016. The results of a Hierarchical linear model indicate that all predictors significantly influenced PDM; PSS positively and cultural values negatively. When the gender of managers and employees is considered, the findings suggest that PSS has a larger impact on PDM when male managers address female employees. Regarding the moderating effect of PSS on cultural values, it is shown that masculinity and power distance lose importance when employees have the support of their supervisors.

## Introduction

Competitiveness, the uncertainty of the economic environment, and socio-cultural changes have resulted in modern, diverse and flexible organizations. In this context, new management styles characterized by the participation of employees in the decision-making process have emerged.

There is a growing consensus among HR researchers and professionals that a participative environment can enhance employees’ job satisfaction, commitment, motivation and productivity (Zhu et al., 2015; Goñi-Legaz and Ollo-López, 2017) and create innovative workplaces, which are beneficial for both employees and employers (Wouters and Wilderom, 2008). Participative management is known by many names, including shared leadership, employee empowerment, employee involvement, participative decision making (PDM), dispersed leadership, open-book management, and industrial democracy (Steinheider et al., 2006). This study focuses on PDM, which can be defined as the extent to which employers allow or encourage employees to share or participate in organizational decision making (Probst, 2005). There are basically two common forms (direct and indirect) of employee PDM that have been widely discussed in the literature (Heller et al., 1998; Markey et al., 2001; Harley et al., 2005). This article focuses on direct employee PDM, which refers to the participation of an individual or group of employees in the decision-making process in the workplace (Bratton and Gold, 2003). The effects of direct employee PDM have been studied extensively in the last few decades (Wallace, 1995; Jones and Kato, 2003; Cox et al., 2006; Boxall and Purcell, 2010). However, the analysis of their determinants has begun to attract relevance only recently (Esser and Olsen, 2011; Gallie, 2013; Lopes et al., 2017). Precisely one of the purpose of this study is to measure the employee PDM in European companies and to advance in the identification and understanding of the internal and external factors that influence direct employee PDM.

### Does the Gender Affect to PDM?

The role of the manager as a starting point for the inclusion of employees in PMD has been demonstrated (Tangirala and Ramanujam, 2012). Previous studies have found a significant relationship between perceived supervisor support (PSS) and employee PDM (Allen, 1992; Hutchison, 1997; Langan-Fox et al., 2002; Allen et al., 2003; Reeves et al., 2012). Therefore, this study tries to ratify whether this relationship is currently valid in the European context. In addition, we are interested in analyzing this relationship from a gender perspective because studies on this topic remain limited (Shaed and Ishak, 2015). Although it seems male and female leaders can differ in how they promote direct employee PDM, the findings are not conclusive. For example, Eagly and Johnson (1990) suggest that females managers tend to promote a participative environment. There are also studies that conclude that women show more supportive behavior toward the programs that have been introduced by their organization compared to men (Collom, 2000). In contrast, some studies, such as those conducted by Sukirno and Siengthai (2011), conclude no significant difference between gender and participation. Beyond managerial stereotypes, some studies have analyzed gender from employee perspectives. Some of them confirm significant differences between men and women in participation (Olorunsola and Olayemi, 2011; Miller, 2012; Sarafidou and Chatziioannidis, 2013). Most of these studies find that women participate less in decision making than men (Markey et al., 2002; De Acedo Lizárraga et al., 2007; Chalchissa and Emnet, 2013). Therefore, one of the main objectives and contributions of this study is the analysis of the influence of the gender of both managers and employees in direct employee PDM, aiming to deepen the research on this topic and clarify the confusion aroused by previous studies.

### How Cultural Values Affect to PDM?

It is known that national cultural values have an influence over organizations (Hofstede, 1984). This paper explains the relation between direct employee participation and two of cultural dimensions identified by Hofstede (1984), power distance and masculinity. Power distance is defined as “the extent to which the less powerful members of the institutions and organizations of a country expect and accept that power is unevenly distributed” (Hofstede, 1984). The literature shows that there are societal cultural differences in how individuals behave in situations of power difference (Rao and Pearce, 2016). In countries of high power distance, employees are afraid to express opinions that differ from those of managers. Several authors have argued that in organizations with a high level of power distance, employees tend to be passive in the decision-making process (Khatri, 2009). In contrast, a low power distance means there is a small distance between employees and manager such that they collaborate at work and have a preference for making decisions throughout consultation.

Most relevant studies on cultural values and participation focus on power distance and uncertainty avoidance. In contrast, few empirical studies have been done on masculinity and employee participation. Assuming the relevance of gender, the current study aims to fill this gap in the literature. Masculinity, one of the culture dimensions studied by Hofstede, is a value that affects the relationship between roles at work and gender. Women are warm, sensitive, and caring in their domestic role, even though they are increasingly demanding occupational roles. Correspondingly, men are considered assertive and competitive, and they tend to demand leadership roles (Bosak et al., 2018). Consistent with this principle, direct employee PDM could be different according to the feminine or masculine culture of the country where companies are located.

The analysis of the direct influence of cultural values in the PDM, as well as the moderating role of the PSS in this relationship, is another contribution of this paper. In addition, this allows identifying which European countries promote more (and less) the direct employee participation in making decisions and comparing this ranking with that regarding the analyzed culture dimensions of Hofstede.

The empirical analysis is based upon the Sixth European Working Conditions Survey (EWCS; Eurofound, 2016) carried out on nearly 44,000 workers belonging to organizations located in 35 European countries. (A more detailed explanation will be offered at 3.1).

The article is structured as follows. After the introduction, the second section defines the concept of direct employee participation (PDM). The third section provides the theoretical arguments that ground the expectations and hypotheses on PDM’ micro- and macro-level determinants. The data and the definition of the dependent and independent variables of the fitted hierarchical regression model are included in the fourth section. Next, the fifth section analyses the results, and, finally, the sixth section includes the discussion of the results and the limitations of the study.

## Literature Review and Hypothesis

### Direct Employee Participation in Decision Making Process (PDM)

Employee participation includes all the practices developed in organizations that allow employees to participate in the decision-making process (Gónzalez, 2009; Miller, 2012). It can take the form of a variety of management practices, such as participative management, empowerment and employee involvement. Moreover, participation may be direct or indirect (Markey et al., 2001; Harley et al., 2005). Indirect employee participation is the involvement of employee representatives in decision-making processes, whereas direct employee participation describes direct interaction between managers and employees. Therefore, direct participation involves the employees themselves, whereas indirect participation occurs through an intermediary of employee representative bodies, such as work councils or trade unions (Cabrera et al., 2003). According to the definition of Eurofound (2015), direct participation can be defined as “opportunities which management provide, or initiatives to which they lend their support, at workplace level, for consultation with and/or delegation of responsibilities and authority for decision making to their subordinates either as individuals or as groups of employees, relating to the immediate work tasks, work organization and/or working conditions.” Hence, this type of participation is encouraged by managers, whereas indirect participation is related to involvement in decisions through representative organisms that are selected by employees (Wilkinson et al., 2010). The current study focuses on direct employee PDM and, consequently, on the influence of managers’ attitudes in that participation.

### Direct Employee PDM and Perceived Supervisor Support (PSS)

Decision-making *per se* is a process linked to authority and power in organizations, so whoever has the power is the person who ultimately exercises decision-making. According to Martín and Quintanilla (1999), participation encompasses the different ways in which power is distributed in an organization, understanding power as the ability to exert influence. In autocratic organizations in which managers exercise monitoring and control over employees, managers exercise decision-making power. Fear of losing that power and distrust of employees’ criteria for differentiating personal goals from organizational ones are the main reasons why managers do not allow employees to make decisions (Mishra and Morrissey, 1990). Over time, organizations have evolved into participatory models in which managers involve employees in decision-making through the distribution of power.

The main driver of this change is trust. As Spreitzer and Mishra (1999) conclude, trust has replaced older models of supervision and control, causing managers to involve employees in the company’s decision-making. It is precisely in this trusted environment where the manager believes that employees decide on organizational goals with the basis of being competent and making good decisions (Morgan and Zeffane, 2003). In addition, Tan and Tan (2000) recognize perceived organizational support (POS) as a precedent for trust, which marks the relationship between employees and the organization. POS theory broadly analyses the relationship between employees’ global beliefs and the organization (Eisenberger et al., 2002). When employees are involved in the decision-making process, they perceive organizational support (Wayne et al., 2002; Park, 2015; Ding and Shen, 2017). Accordingly, employee participation can be perceived as a sign of organizational commitment to employees. In fact, Patriota (2009) explains that employee participation is directly or indirectly related to POS. POS and PSS are concepts that are positively associated (Armstrong-Stassen and Cameron, 2003). Given that supervisors represent organizations, employees could POS from their supervisor. As reported by Maertz et al. (2007), employees build their relationship with managers on the basis of how they value their contributions. In this respect, previous findings reveal a positive relationship between PSS and trust in supervisors (Stinglhamber et al., 2006).

Simultaneously, based on social exchange theory, employees value recognition and fair treatment, whereas supervisors value performance and loyalty (Paillé et al., 2013). Given that affirmation, the supervisor plays a crucial role in participation if the leader-member exchange (LMX) approach is considered. Erdogan and Enders (2007) points that LMX is the most appropriate theory to explain how leaders and supervisors influence employees. This theory assumes that managers create different kinds of relationships with subordinates in terms of an exchange (Meiners and Boster, 2012). In high LMX, supervisors create social exchanges with trust, whereas lower LMX is featured by a minimal level of support. Previous researchers observe that this supervisor-subordinate combination has a positive impact on employee involvement in participation (Torka et al., 2010).

Therefore, PSS is considered by the current study as a micro variable that determines direct employee participation.

As a result, the following is expected:

H1: PSS by employees is positively related to their direct PDM.

### Employee PDM and Gender

The gender perspective is included in this study at two different points of view. On one hand, direct PDM is analyzed from the perspective of the employees’ gender. This analysis will allow us to verify whether the participation of women in making decisions at workplace is lower than that concerning men, which would corroborate the existence of a situation of inequality even in the 21st century. This measurement of inequality between men and women is a novelty in the literature and one of the contributions of this study. The gender gap in a workplace context is usually related to differences in wages (gender pay gap) (Ziderman, 2014) and the number of women in decision-making positions and is not often related to the level of direct participation in making decisions. This leads to the formulation of the second hypothesis:

H2: Female employees have lower direct PDM than men.

On the other hand, the influence of the manager’s gender is studied assuming its role as a promoter of direct employee participation. This perspective is also of interest for two main reasons. The first is the relevant papers on women in management. According to a study from Eurostat (2017), in the European Union, women continue assuming responsibility role in organizations (although the number of women in management positions is still lower than men). Second, conforming to Cuadrado et al. (2015) female managers often acquire participatory and democratic management styles compared to men. The creation of a participative environment is typical of transformational leadership (Zulfqar et al., 2016). It means a transformational leader promotes the involvement of employees in the decision-making process. Some authors reason that a transformational leadership style is associated with the female gender role (Pounder and Coleman, 2002; Wolfram and Gratton, 2014). In this sense, according to Druskat (1994), women value connection, collaboration and discussion, concluding that female managers exhibit significantly more transformational leadership behaviors and significantly fewer transactional leadership behaviors than male leaders. Consistent with this affirmation, female managers lead in a participatory manner and encourage others to participate in decision making. Concerning the characteristics of managers toward participation, women are considered as a paternalistic figure that protects and creates circles of trust and collaboration among employees (Melero, 2011). In addition, female manager are expected to be more inclusive and encourage employees to express their ideas. This feature gives rise to a third hypothesis:

H3: Employees managed by women have a higher direct PDM than those managed by men.

Previous studies analyze the relationship between managers and employees in terms of gender. O’Leary and Ryan (1994) found that women employees expect their female managers to be more understanding and more giving than men. Likewise, a study by Cuadrado et al. (2015) shows that female employees tended to rate their female managers as more effective. Similarly, they found that the gender dyad association between employees and managers was stronger amongst females versus males. In contrast, other research points to the queen bee phenomenon. This is a derogatory label given to women managers. According to this idea, women managers pursue individual success in male-dominated work settings and tend to create distance with other woman peers (Kanter, 1987; Derks et al., 2016). In other words, women managers do not support women employees in particular. To shed light on this opposite ideas, this paper explores how participation is affected by PSS according to the manager-employee gender dyad composition.

H4: Perceived Supervisor Support (PSS) has a different impact on direct employee PDM according to the manager-employee gender dyad.

### Direct Employee PDM and National Cultural Values: External Context

Research on cross-cultural management (Gelfand et al., 2007; Tsui et al., 2007) indicates that the extent to which people in organizations hold cultural values and beliefs determines the way in which employees react to aspects of their work. According to Kroeber and Kluckhohn (1952), “culture consists of patterns, explicit and implicit, of and for behavior acquired and transmitted by symbols, constituting the distinctive achievements of human groups, including their embodiments in artifacts; the essential core of culture consists of traditional (i.e., historically derived and selected) ideas and especially their attached values” (p. 181). Sagie and Aycan (2003) suggest that culture explains and determines reason, essence, issues and people involved in the decision. The latter two points are critical for this study. The same authors explain that culture determines the way to understand PDM for both employees and managers. From the wide range of cultural dimensions studied by Hofstede, this framework is based on two: power distance and masculinity/feminity.

Power distance is defined as “the extent to which the less powerful members of the institutions and organizations of a country expect and accept that power is unevenly distributed” (Hofstede, 1984). That is, individuals of a culture with a small distance to power recognize working in decentralized organizations where they are consulted by their superiors and treated as equals (Ortega et al., 2001). In contrast, countries with a high power distance have a very centralized leadership and have a very autocratic management style (Hofstede, 1984) that leaves little autonomy to workers. Thus, a large distance to power corresponds to organizations in which the number of hierarchical levels is significant (Sui Pheng and Yuquan, 2002), since they form the basis of formal power. Jaeger (1986) notes that the high distance between hierarchical levels sometimes causes individuals of different levels to resist working together, thereby conditioning the creation of work teams. The aforementioned hierarchical differentiation causes the communication flow to occur only vertically and downstream from the dome to the lower levels.

In organizations with a low power distance, communication between manager and employee is more open, which translates into a high level of participation and therefore joint decision-making. This is because employees of low-power organizations are more likely to have direct contact with their managers. Conversely, if the distance becomes greater, employees will work independently of the managers. With this, contact is reduced, and the possibilities of co-decision tend to decrease (Białas, 2009). Furthermore, previous researchers have indicated that women tend to see the world and organizations as a flat structure, whereas men identify hierarchical relationships (Ayman et al., 2009).

Following gender differentiation, countries with a high level of masculinity make differentiation between male and female responsibilities. In this sense, women are related to house responsibilities and men are able to develop a career (Hofstede et al., 2008). Masculinity values assume that gender has an impact in the workplace. According to Newman and Nollen (1996) in male organizations, men are primarily interested in performance, profits and recognition; in women, there is an interest in relationships. Hofstede (1984) explains that women tend to “humanize” the job, promoting cooperation. Additionally, aggressiveness is the most acknowledged feature in highly masculinity-oriented companies (Ohlsson and Odelj, 2007). In this sense, it could be said that female organizations promote a more participative environment than male organizations.

The present study aims to understand if direct employee PDM promoted by organizations is linked to the cultural dimensions of the country in which they are located. Thus, the following hypotheses are established:

H5: The greater the power distance in the culture where the organization is located, the lower the level of direct employee PDM.

H6: The higher the level of masculinity in the culture where the organizations is located, the lower the level of direct employee PDM.

In the literature, some studies analyze the cultural values at the individual level as a moderator variable of POS (Farh et al., 2007). Considering that the dimensions described above affect organizational practices in a general way, this paper goes one step ahead by trying to demonstrate that PSS is a micro variable that corrects the impact from cultural dimensions.

A study on the Chinese hotel industry (Humborstad et al., 2008) indicates that perceived supervisor and organizational support (between other variables) act as a moderator between empowerment and the quality of delivery service in a traditionally high-power-distance culture. Moreover, Park (2015) demonstrated that POS mediated the relationship between employee participation and organizational commitment. Considering the moderator effect of PSS, this research attempts to evaluate the corrector effect of PSS over power distance.

H7: Perceived supervisor Support has a moderating effect on the influence of the power distance dimension on direct employee PDM.

Apparently, women and men are very similar in their leadership styles, but females often have a larger supporting role (Gregory, 1990). The gender roles can then account for the variation in the use of PSS and its relation with masculinity/feminity values, but previous research has not addressed this issue. From this side, it is expected that the gender of the supervisor providing support has an effect on prior values where the organization is located.

H8: Perceived supervisor support has a moderating effect on the influence of the masculinity dimension on direct employee PDM.

## Data and Summary Statistics

The testing of previously formulated hypotheses involved a three-stage process. First, a factor analysis was carried out to obtain the values of the variables related to employees (direct PDM and PSS). Second, descriptive and inferential statistics analysis were carried out in order to justify the selection of the variables used to predict the PDM. In addition, thirdly, hierarchical linear modeling (HLM) was applied to explain direct employee PDM by PSS at a first level (employee) and the national cultural values (Power distance and Masculinity) at a second level (country). The gender of the employees and their managers was included in the analysis as a control variable. The data analysis procedure was conducted using the 25th version of SPSS statistic software.

### Definition of the Sample

The analyses are based upon the Sixth European Working Conditions Survey (EWCS; Eurofound, 2016) carried out by Eurofound. Nearly 44,000 workers were interviewed in 35 countries: the 28 EU Member States, the five EU candidate countries, and Norway and Switzerland. Its findings provide detailed information of issues such as physical and psychosocial risks, work organization, work–life balance, and health and well-being. In each country, a multistage stratified random sampling design was used.

Since the objective of this study is to analyze direct PDM by employees (no managers), as well as the role of their supervisors’ supporting in this participation, the cases related to managers were identified and later removed from the original database. The final sample includes 26,079 employees with information about the gender of them and of their bosses. According to the contingency table (Table 1), the proportion of male and female employees is practically the same (50.4 and 49.6%). However, the gender of their managers is mostly male (68.2% of male managers vs. 31.70% of female managers). A hypothesis test was carried out to ratify that proportion of men in managerial positions is significantly greater than that of women (*p*-value = 0.000 when testing the null hypothesis that the proportion of men in management positions is 50% against the alternative that it is greater). These figures are close to the information published by Eurostat (2017), extracted from the four-yearly structure of earnings survey for reference year 2014. According to these statistics, at the EU level, approximately a third (35%) of managers are women. This figure denotes that there is still a way to go to achieve equal opportunities for men and women in the workplace in the European context.

**Table 1:** Contingency table of gender dyad (manager-employee) composition.

		Gender_employee		Total
		Male	Female	
Gender_manager	Male	11,530	6,294	17,824 (68%)
	Female	1,617	6,638	8,255 (32%)
Total		13,147 (50.4%)	12,932 (49.6%)	26,079 (100%)


If the gender of both employees and their managers is considered, 65% of male managers work with male employees (so only 35% of them manage female employees). Regarding employees managed by women, only 19% are men (compared to 80% of women). Therefore, in most of the cases observed, the gender of employees and bosses coincides. This relationship was ratified with a Chi-square test (*p*-value = 0.000, so the null hypothesis of independence between the gender of employees and their managers was rejected). Moreover Phi coefficient was computed (0.42) showing a moderate and significant (sig. = 0.000) relationship between both genders.

### Measurement of Variables

#### Dependent Variable: Direct Employee PDM

The survey contains several relevant questions providing information about direct employee participation in making decision (PDM). Concretely, employees are asked about eight questions related to their participation in making decisions about the order of their tasks, their methods of work, the speed or rate of work, improving the work organization, choosing their work colleagues, applying their own ideas, influencing important decisions and being consulted before objectives are set. Cronbach’s alpha was computed in order to measure the internal consistency of these eight items used to measure the construct “Participation.” The value of alpha was 0.814 so it ratifies a good internal consistency. In addition, the Cronbach alpha coefficient was reduced if any of the elements considered was removed from the reliability analysis. A factor analysis was carried out based on these eight survey questions, specifically, principal component analysis. The results ratified that factorial analysis was appropriated (Bartlett’s Test of Sphericity, sig. 0.00, and Kaiser-Meyer-Olkin Measure of Sampling Adequacy 0.864, so greater than 0.75). Two components were found that explained 63% of the total variance. This fact ratifies the theoretical classification of direct PDM into two categories: individual task discretion and organizational participation (Inanc et al., 2015). The first component (which explains 36% of the total variance) is associated with employee involvement in the work organization, the possibility of applying their own ideas and the influence in important decisions, that is, the organizational level. The second component (27% of the total variance) is associated with how employees can make decision related to his/her own work, that is, individual task discretion. The scores of each component for each participant were obtained by the regression method. Thus, the linear combination of these factor scores weighted by their contribution to the variance became the final value of the dependent variable. Therefore, this study defines the direct employee PDM construct over both concepts (strategic and operational levels).

#### Independent Variables: PSS and National Cultural Values

Perceived supervisor support by interviewed employees was considered the independent variable in the hierarchical regression model in the first stage at the company level. Later, at the country level, indexes regarding power distance and masculinity were added.

Perceived supervisor support can be defined as employees’ general opinion concerning the degree to which supervisors value their contributions and care about their well-being (Eisenberger et al., 2002). It was measured through factor analysis, using six questions of the survey related to this aspect. Employees were asked if they received respect, praise and recognition, useful feedback, encouragement, support and help from their boss. In this case, only one component explained 63% of the variance. The Cronbach alpha coefficient was 0.878, so there is a good internal consistency of the six items used to measure the dimension “Perceived Supervisor Support.” In addition, this coefficient was reduced if any of the elements considered was removed from the reliability analysis.

For the country level, the indexes of the cultural values of power distance and masculinity provided by Hofstede (1984) were considered^1^.

#### Control Variable: The Gender

Several authors have suggested that the gender of one’s boss is an important control variable in direct participation research (Gregory, 1990; Cuadrado et al., 2015). It has been argued previously (see section “Employee PDM and Gender”) that the gender of the boss defines the leadership style (Newman and Nollen, 1996; Ohlsson and Odelj, 2007; Melero, 2011). Therefore, this paper considers the effect of gender in employee participation. In particular, the gender of both employees and managers was included as control variables in the regression model to explain the relationship between the variables previously described.

### Descriptive and Inferential Analysis

Direct employee PDM and PSS values obtained by factor analysis were ranked between 0 and 1 to make its interpretation easier. Descriptive statistics (Mean and Standard Deviation) for both variables are reported in Table 2, as well as the results of an inferential analysis (independent sample *t*-test).

**Table 2:** Descriptive statistics and hypothesis testing according to the gender of employees.

	Female employees		Male employees		Levene’s test	*t*-test	Decision
	Mean	SD	Mean	SD	*p*-value	*p*-value	
Direct employee PDM	0.5528	0.3073	0.5826	0.31121	0.228	0.000	Significant difference
Perceived supervisor support (PSS)	0.6847	0.1816	0.6758	0.17545	0.000	0.007	Significant difference


For example, regarding direct employee PDM, the statistics indicate that female employees participate less, on average, than male employees (0.5528 vs. 0.5826). After the acceptance of equality of the variances (Levene’s test *p*-value is 0.228), the null hypothesis of equality of the two means of male and female employee PDM is rejected (*p*-value = 0.000). Therefore, female employees have lower direct PDM than male employees (the confidence interval for the difference between females and males ranges from -0.037 to -0.022). This result supports hypothesis *H2*, which predicts a lower direct PDM from women employees. Concerning the values of PSS, female employees perceive greater support from her supervisor (0.6847), on average, than men (0.6785). In this case, the *t*-test ratifies this difference (*p*-value 0.007) under the assumption of inequality of the variances (Levene’s test *p*-value 0.000). Therefore, if women participate less but perceive greater support from their supervisor, the higher PSS perceived by female employees does not always translate into higher direct PDM. This result highlights the need to delve into the relationship between PSS, PDM and gender through regression analysis.

When the gender of managers is analyzed without consider the gender of the employees (see Table 3) direct employee PDM is slightly greater for those cases managed by women, but the difference is not significant (hypothesis *H3* is not supported by data). According to PSS, it was significantly greater for employees with female managers.

**Table 3:** Descriptive statistics and hypothesis testing according to the gender of managers.

	Female managers		Male managers		Levene’s test	*t*-test	Decision
	Mean	SD	Mean	SD	*p*-value	*p*-value	
Direct employee PDM	0.5440	0.2993	0.5411	0.3020	0.154	0.489	Non-significant difference
Perceived supervisor support (PSS)	0.6913	0.1786	0.6772	0.1780	0.000	0.007	Significant difference


In order to clarify the influence of the interaction between the genders of both, managers and employees, in PDM, a *t*-test for independent samples was carried out considering all the possible gender combinations (Table 4). All the differences on PDM means were significant, except that regarding female employees managed by men and those managed by women (perhaps due to the smaller sample size of the group representing female manager-female employee). This analysis ratified the higher participation of men employees and the greater PDM mean for those employees (male and female) managed by women.

**Table 4:** *t*-test of differences between PDM means according to gender dyad composition.

Case	Gender combination (Manager-employee)	PDM mean	Sig. level	Confidence interval
1	Male-male	0.5114	0.002	0.0050; 0.0226
2	Male-female	0.4975		
3	Female-male	0.5303	0.001	0.0112; 0.0419
4	Female-female	0.5039		
5	Male-female	0.4975	0.205	–0.0160; 0.0034
6	Female-female	0.5039		
7	Female-male	0.5303	0.012	0.0042; 0.0335
8	Male-male	0.5114		
9	Male-female	0.4975	0.000	–0.0485;-0.0170
10	Female-male	0.5303		


Therefore, direct employee PDM (dependent variable) is different according to the gender of both employees and managers. This result justifies the inclusion of gender as a control variable that influences the relationship between PDM and PSS.

Moreover, a correlation analysis was analyzed to justify the inclusion of the independent variables in the regression model (see Table 5) as well as to detect if there is a problem of multicollinearity. The results indicate that all the correlation coefficients are significant. Direct employee PDM is positively correlated with PSS. On the contrary, PDM was negatively correlated with national cultural values. In this way, the larger the power distance level of the country where the company is located, the less direct employee PDM. A similar trend exists for the masculinity dimension: the more masculine the country, the less direct employee PDM. The correlation between the independent variables is low (near 0), so it seems there is not a problem of multicollinearity (low values of VIF ratify this fact after with the regression model).

**Table 5:** Linear correlation coefficients between PDM and the explanatory variables.

Variable	1	2	3	4
1. Direct employee PDM	1			
2. Power distance	–0.091**	1		
3. Masculinity	–0.083**	0.072**	1	
4. PSS	0.449**	–0.028**	–0.049**	1


^∗∗^Significance level 5%.

To study in deep the influence of the gender combination (manager-employee) in the linear relation between PSS and PDM, an analysis of linear correlation coefficient was made for each subgroup. Then a hypothesis test about the difference of these coefficients was carried out using Fisher’s Z transformation in order to detect if these differences were significant or not (Table 6). Results showed significant differences in the strength of the association between PDM and PSS because of the gender combination (manager-employee). Concretely, the strongest relationship happens between male managers and female employees. That if the direct association between the PSS and PDM is highest when a female employee perceived the support of her male manager.

**Table 6:** Hypothesis test about the difference between linear correlation coefficients of PDM and PSS according to gender dyad composition (Fisher’s Z transformation).

Gender combination (manager-employee)	Difference r_PSS_PDM_ by gender (r_i_–r_j_)	Z(r)	Decision
Male-male r_1_	0.436–0.476	–3.22283593	H_1_ (significant difference)
Male-female r_2_			
Male-male r_1_	0.436–0.439	–0.13960063	H_0_ (no significant difference)
Female-male r_3_			
Male-male r_1_	0.436–0.453	–1.37489636	H_0_ (no significant difference)
Female-female r_4_			
Male-female r_2_	0.476–0.439	1.677579713	H_1_ (significant difference)
Female-male r_3_			
Male-female r_2_	0.476–0.453	1.666780855	H_1_ (significant difference)
Female-female r_4_			
Female-male r_3_	0.439–0.453	–0.62970799	H_0_ (no significant difference)
Female-female r_4_			


## Results

As the predictor variables are at two different hierarchical levels (PSS at the employee level, and the national culture indexes at the country level), HLM was applied to analyze variance in direct employee PDM using the whole sample (see Table 7 Model 1). Due to the interest in analyzing the influence of gender in the relationship studied, and after detecting differences in the linear association between PSS and PDM variables according to the gender dyad composition, the regression model was applied over different subsamples. Concretely, Models 2 and 5 are associated with those samples with female and male managers, respectively (without distinguishing the gender of the employees). Models 3 and 4 are related to the female manager sample, but separated by the gender of the employees. The same occurs with models 6 and 7, but in this case for the sample of male managers.

**Table 7:** Results of the hierarchical regression models: direct employee participation (Standardized β-values).

Variables	Total Sample		Female manager			Male manager		
		Model 1	Model 2	Model 3	Model 4	Model 5	Model 6	Model 7
	
			Total employee	Male employee	Female employee	Total employee	Male employee	Female employee
**Step 1: Independent variable**								
PSS		0.448***	0.442***	0.434***	0.445***	0.451***	0.435***	0.480***
**Step 2: Independent variable + Main effects**								
PSS		0.443***	0.435***	0.429***	0.438***	0.446***	0.430***	0.474***
Masculinity		–0.063***	–0.053***	–0.038	–0.054***	–0.069***	–0.074***	–0.058***
Power distance		–0.080***	–0.090***	–0.064***	–0.095***	–0.077***	–0.080***	–0.073***
ΔR^2^		5.47%	6.67%	3.17%	7.07%	5.39%	6.35%	3.91%
**Step 3: Independent + Main effects + Mediating effects**								
PSS		0.444***	0.439***	0.430***	0.442***	0.446***	0.430***	0.474***
Masculinity		–0.061***	–0.052***	–0.038	–0.053***	–0.066***	–0.072***	–0.054***
Power distance		–0.079***	–0.089***	–0.064*	–0.094***	–0.076***	–0.080***	–0.070***
Masculinity + PSS		0.021**	0.023*	0.007	0.026*	0.0019**	0.017	0.023
Power distance + PSS		0.012*	0.020	–0.007	0.027*	0.007	0.002	0.017
ΔR^2^		0.47%	0.48%	0%	0.94%	0%	0.50%	0.42%
Final adjusted R^2^		21.2%	20.91%	19.2%	21.3%	21.5%	20.1%	23.9%


*^∗∗∗^*p* < 0.001, ^∗∗^*p* < 0.01, ^∗^*p* < 0.05.*

The standardized values of the regression coefficients (showed in Table 7) suggest that all predictors are significant.

As expected in Hypothesis 1, and according to the correlation coefficient previously computed (Table 5), PSS is positively related to direct employee PDM. Moreover, the findings suggest again that PSS (Model 7: β = 0.474^∗∗∗^) has a higher impact on employee participation when male managers address female employees. The difference between this coefficient and the rest of the cases would be significant according to the results of the hypothesis test of the difference between correlations coefficients previously made (Table 6). In this sense, Hypothesis 4 is supported, due to PSS having a different impact on direct employee PDM according to the manager-employee gender dyads. Regarding cultural values, both power distance and masculinity have a significant negative relationship with each outcome variable. Thus, in organizations located in countries with a high level of masculinity and power distance, participation is less promoted. These two results reinforce strong support for Hypothesis 5 and 6. However, masculinity (Model 3: β = -0.38) loses significance when women assume a management position. Regarding the moderating effect, Hypothesis 8 is supported. The interaction between PSS and masculinity has a significant and positive relation with PDM, which means PSS balances the negative influence of masculinity over PDM (Model 1: β = 0.021^∗∗^; Model 2: β = 0.023^∗^; Model 4: β = 0.026^∗^; Model 5: β = 0.019^∗∗^); however, it has no significant relationship when the manager is male. Thus, PSS moderates masculinity level in organizations where managers are women.

**FIGURE 1:**
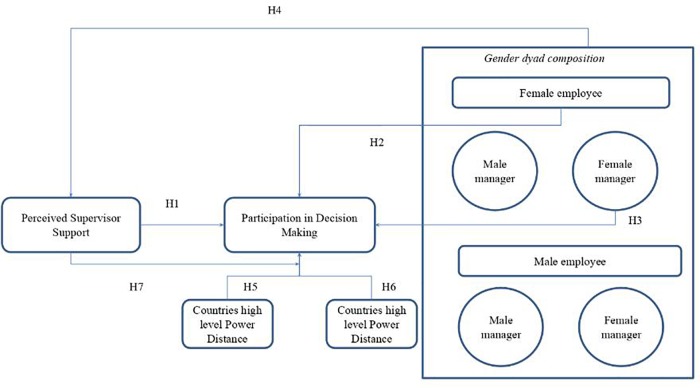
Relations between variables and hypothesis.

In addition, PSS has a low moderating effect in power distance in the general model (β = 0.12^∗^), and loses significance in the gender classification (Model 2: β = 0.020; Model 3: β = -0.007; Model 5: β = 0.007; Model 6: β = 0.002; Model 7: β = 0.017). The moderating effect of PSS over power distance means Hypothesis 7 is not totally support. Only when female managers lead female employees is the moderating effect significant. The relationship between the analyzed variables and the tested hypothesis are included in Figure 1.

In order to deepen the analysis by countries, a ranking was made according to their average values in direct employee PDM. This classification was compared with the ranking of countries according to their cultural values (see Table 8). This analysis ratifies that those countries with the highest values in direct employee PDM (first positions in this ranking) such as Finland, Denmark, Norway, Ireland, and Netherlands, have also the lowest rates of masculinity and power distance (the last positions in these rankings). On the contrary, it observes that countries such as Spain, Portugal, Greece, Italy, and Oriental countries (Bulgaria, Slovakia, or Lithuania) present low values in terms of participation, and are featured by high levels of power distance and masculinity.

**Table 8:** Ranking of countries according to their direct employee PDM (average) and cultural values.

Country	Sample proportion	PDM Mean	Masculinity index	Power distance index	Ranking by participation	Ranking by masculinity	Ranking by power_distance
Finland	2.70%	0.686	26	33	1	25	26
Denmark	2.08%	0.683	16	18	2	28	30
Norway	2.34%	0.668	8	31	3	30	27
Ireland	2.40%	0.664	68	28	4	7	29
Netherlands	2.48%	0.663	14	38	5	29	22
Malta	2.21%	0.640	47	56	6	15	17
Slovenia	4.23%	0.637	19	71	7	26	7
Sweden	2.28%	0.621	5	31	8	31	28
United Kingdom	3.65%	0.615	66	35	9	8	23
Austria	2.74%	0.607	79	11	10	4	31
Turkey	5.06%	0.592	45	66	11	16	11
Belgium	6.36%	0.590	54	65	12	13	12
Estonia	2.37%	0.589	30	40	13	24	21
Switzerland	2.84%	0.583	70	34	14	5	25
Czech Republic	2.84%	0.579	57	57	15	11	15
Romania	2.67%	0.578	42	90	16	19	3
Luxembourg	2.24%	0.577	50	40	17	14	20
France	4.17%	0.574	43	68	18	17	10
Hungary	2.92%	0.565	88	46	19	2	19
Spain	8.83%	0.563	42	57	20	20	16
Poland	3.01%	0.546	64	68	21	10	9
Albania	1.68%	0.542	80	90	22	3	2
Serbia	2.53%	0.530	43	86	23	18	4
Italy	3.42%	0.517	70	50	24	6	18
Croatia	2.79%	0.513	40	73	25	21	6
Greece	2.36%	0.508	57	60	26	12	14
Bulgaria	2.85%	0.496	40	70	27	22	8
Portugal	2.32%	0.487	31	63	28	23	13
Germany	6.30%	0.475	66	35	29	9	24
Slovakia	2.87%	0.472	100	100	30	1	1
Lithuania	2.43%	0.4675	19	75	31	27	5


## Discussion and Conclusion

This research pursued a double objective. On the one hand, to know the level of direct participation in decision-making by employees in European companies, in the current period (second decade of the 21st century). On the other hand, to further clarify the debate on the effect of PSS in the aforementioned participation, as well as the effect of national cultural values and the gender of both managers and employees in such a relationship.

Concerning the first objective, the study reveals that currently, in the European context, the number of women in management positions is still considerably lower than that of men. In addition, direct participation in decision-making is also lower in the case of women. Therefore, we can affirm that today, in the European company, women are still in a situation of inequality with respect to men in terms of participation in decision-making and in the performance of leadership positions.

Regarding the second purpose, the research shows that PSS has a direct relationship with employee PDM, whereas cultural dimensions such as masculinity and power distance have a negative effect. Additionally, the current study confirms that the gender of managers affects direct employee PDM in different ways based on the gender of the employee. Concretely, direct employee PDM is, on average, greater in the case of female managers, especially with male employees (the higher PDM of women employee managed by women managers was not significant, perhaps due to the smaller proportion of this subset in the whole sample) but the difference is not significant. It has been found, that PSS has a greater influence on direct PDM when the manager is a man and the employee is a woman. Therefore, when a male manager gives his support to a female employee, this support is transformed into a higher PDM than in other gender combinations.

It has also been found that female managers tend to mitigate the effect of the masculinity of the country where the organization is located.

Previous researchers suggest that a participative environment can enhance employees’ job satisfaction, commitment, motivation and productivity (Zhu et al., 2015; Goñi-Legaz and Ollo-López, 2017). Managers contribute to a participative environment by promoting collaboration among employees. For this reason, managers play a crucial role in motivating employees to participate in PDM. Consistent with this, the cultural values of the countries where organizations are located affect direct employee PDM (Sagie and Aycan, 2003).

In general, all hypotheses were supported. Consistent with previous research, PSS plays a crucial role as a promoter of employee participation in organizations. These results lend further support to the notion that employees feel supported by managers when they encourage them to participate in the decision-making process (Wayne et al., 2002; Torka et al., 2010; Park, 2015). Evidence from this study confirms that the so-called dyad gender composition of both managers and employees has an effect on participation (Ayman et al., 2009). This study reinforces the results of previous findings: men and women have significantly different orientations leading to gender differences in the effects of participation initiatives (Olorunsola and Olayemi, 2011; Miller, 2012; Gallie, 2013; Sarafidou and Chatziioannidis, 2013). In this respect, male supervisor support has a positive and high influence when dealing with female employees. And this is one of the main findings of this research.

Women in management position are also more supportive in organizations located in countries where the masculinity value is predominating; they are considered agents of change for gender integration (Ranganathan, 2016). In other words, the PSS provided by female managers influences positively in the PDM, overall in countries with high level of masculinity, This constitutes another important contribution of the present study.

Previous studies proved that PSS delivered more quality service in cultures with traditionally high power distance (Ghosh, 2011). However, contrary to the prediction, there was no significant impact of supervisor support as a moderator for power distance, unless female managers lead female employees. In this situation, in organizations located in countries featuring a higher power distance, female managers tend to mitigate the effect.

Based on these findings, it can be concluded that gender differentiation is a crucial feature in the analysis of participation in Europe organizations.

## Limitations and Future Research

Although previous studies indicated that gender diversity and a balanced number of women and men in PDM can lead to positive outcomes in the organization, inequalities between gender in PDM remain a large concern and an unresolved problem in most companies around the world due to the huge gap between the number of women and men in PDM positions. This could be precisely the cause of one limitation of this study, the smaller sample of female managers in comparison to that of male managers. This could affect the capacity to generalize some of the findings. Additionally, the cases of female employees leaded by female managers are astonishingly lower in comparison with those leaded by male managers. This makes difficult the comparison of the results for both groups.

Another weakness of this study is that it is based solely on the perceptions of the workers, so it would be appropriate to take into account the opinion of the managers and compare the results.

In future research, it would be interesting to analyze in more depth the difference by countries in direct employee PDM as well as the influence of other cultural dimensions proposed by Hofstede not included at this study.

Finally, it is necessary to highlight that right now, this paper has an extraordinary opportunity to promote the role of women in the economic sphere, which is indispensable to entails fostering gender equality in education, employment and leadership positions, so we should take advantage of the moment.

## Author Contributions

All authors listed have made a substantial, direct and intellectual contribution to the work, and approved it for publication.

## Conflict of Interest Statement

The authors declare that the research was conducted in the absence of any commercial or financial relationships that could be construed as a potential conflict of interest.

## Footnotes

1 Visit https://www.hofstede-insights.com/models/national-culture/ for further details about index
